# Genomic characterization and phylogenetic analysis of a clinical *Streptococcus parasuis* isolate from a human patient

**DOI:** 10.3389/fcimb.2026.1759415

**Published:** 2026-06-19

**Authors:** Jiachen Zhao, Haoyuan Jin, Ke Wu, Xiangfeng Dou, Baihui Han, Bing Lv, Fu Li, Changying Lin, Daitao Zhang, Zhaomin Feng, Zhichao Liang

**Affiliations:** 1 Institute of Infectious Diseases and Endemic Diseases Control, Beijing Center for Disease Prevention and Control, Beijing Key Laboratory of Surveillance, Early Warning and Pathogen Research on Emerging Infectious Diseases, Beijing Research Center for Respiratory Infectious Diseases, Beijing, China; 2 Beijing Chaoyang District Center for Disease Prevention and Control, Beijing, China

**Keywords:** antibiotic resistance, phylogenetics, *Streptococcus parasuis*, virulence factors, whole-genome sequencing, zoonosis

## Abstract

**Background:**

*Streptococcus parasuis* is a bacterial species recently reclassified from *Streptococcus suis*. It has been isolated from pigs and cattle, where it may cause various infections. Human infections caused by *S. parasuis* are rare, with fewer than five publications reported human clinical case to date, and current knowledge regarding its pathogenic potential, antibiotic resistance, and evolutionary context in humans is limited.

**Results:**

In this study, the *S. parasuis* strain S62 was isolated from the blood of a febrile patient. Whole-genome sequencing revealed a genome of 1.93 Mb with a G+C content of 39.5%. Phylogenetic and ANI analyses confirmed that S62 belongs to *S. parasuis* and is closely related to strains 7500, 221006, NN1, and BS26. The genome harbors virulence-associated genes, including *hasC*, and putative antibiotic resistance determinants such as a *vanY*-like glycopeptide resistance gene and the *patA–patB* efflux system. Phenotypic antimicrobial susceptibility testing showed that S62 was sensitive to most tested antibiotics, with intermediate susceptibility observed only for tetracycline and clindamycin. Functional annotation highlighted the strain’s metabolic versatility and potential for environmental adaptation.

**Conclusion:**

This study provides a detailed genomic characterization of a human clinical *S. parasuis* isolate, shedding light on its virulence potential, resistance determinants, and evolutionary relationships. These findings provide a baseline for further investigation and monitoring of *S. parasuis* in human infections.

## 1 Introduction

*Streptococcus suis* (*S. suis*) is a major Gram-positive pathogen in swine, capable of causing severe systemic diseases such as septicemia, meningitis, and arthritis, which result in substantial economic losses to the global swine industry (Neila-Ibáñez et al., 2021; Rayanakorn et al., 2020; Salvador et al., 2024). Beyond its veterinary impact, *S. suis* also poses a significant zoonotic threat (Feng et al., 2010; Lun et al., 2007). Particularly in Southeast Asia, human cases can occur through occupational exposure or the consumption of undercooked contaminated pork products, leading to life-threatening outcomes such as streptococcal toxic shock syndrome (STSS) (Brizuela et al., 2023; Huong et al., 2014; Weinert et al., 2015). Thus, members of this bacterial group pose significant challenges to both animal health and production, as well as to public health.

S. suis is recognized as an important zoonotic pathogen with thousands of human cases reported worldwide. A systematic review of studies from 1983 to 2017 identified 1,454 confirmed human cases from 32 publications, although the true number is likely higher due to underdiagnosis and limited awareness (Rayanakorn et al., 2018). Another multi-source epidemiological survey in Thailand documented 1,798 human cases between 1987 and 2021 (Kerdsin, 2022).

Recent whole-genome phylogenetic analysis have shown that several lineages previously classified as *S. suis*—particularly serotypes 20, 22, and 26—have now been reclassified as a distinct species, *Streptococcus parasuis* (*S. parasuis*) (Guo et al., 2022). This newly identified species has been isolated from diseased pigs and cattle, where it has been linked to pneumonia, septicemia, and arthritis (Qi et al., 2023).

In contrast, human infection caused by *S. parasuis* remains extremely rare. A PubMed search (accessed January 29, 2026) using the terms “*Streptococcus parasuis*” AND (human OR infection OR meningitis) retrieved 15 records, of which fewer than five publications reported human clinical cases. Recent genomic reports have primarily emerged after 2021, including studies characterizing human- or animal-derived isolates in China and Japan, yet only a small number of complete genomes are publicly available. Consequently, the genetic diversity, host adaptation, and pathogenic mechanisms of *S. parasuis* remain poorly defined.

Given the scarcity of human-derived *S. parasuis* genomic data, the characterization of strain S62 provides a valuable opportunity to investigate the genetic basis of human infection and potential cross-species transmission. In this study, we report the isolation of a clinical *S. parasuis* strain (S62) from the blood culture of a febrile patient. We conducted whole-genome sequencing and comprehensive genomic analysis to investigate its genetic characteristics. Through comparative genomics, virulence and resistance profiling, and phylogenetic reconstruction, this work provides novel insights into the pathogenic potential and evolutionary context of *S. parasuis*. with implications for its zoonotic risk and public health surveillance.

## 2 Case presentation

A 50-year-old woman from Hebei Province, northern China, presented with an acute febrile illness. She had been diagnosed with type 2 diabetes mellitus approximately three weeks prior to disease onset and was receiving dapagliflozin and metformin, with suboptimal glycemic control.

On day 0, the patient developed sudden-onset fever with a maximum body temperature of 39.3 °C, accompanied by chills, fatigue, profuse sweating, chest tightness, back pain, nausea, and vomiting. She initially sought care at a local clinic, where she was treated symptomatically for presumed influenza; however, her fever recurred shortly after transient improvement.

On day 1, the patient was admitted to a county-level hospital. Laboratory tests revealed markedly elevated inflammatory markers, while the white blood cell count was mildly increased. Urinalysis showed abnormalities, and urine culture yielded *Escherichia coli* and *Candida glabrata*. A urinary tract infection was suspected, and the patient received sequential broad-spectrum antibacterial and antifungal therapy. During a two-week hospitalization, her fever resolved, and she was discharged in stable condition.

On day 16, the patient experienced recurrent high-grade fever with symptoms similar to those observed at disease onset. She sought further evaluation at a tertiary care hospital in Beijing. Laboratory investigations demonstrated leukocytosis with neutrophilia and elevated inflammatory markers, while imaging of the urinary system revealed no significant abnormalities. Empirical intravenous antimicrobial therapy was initiated; however, low-grade fever persisted.

On day 18, blood cultures were obtained for further diagnostic evaluation. Several days later, the blood culture yielded a Gram-positive streptococcal isolate, which was initially identified as *S. suis* by routine microbiological testing and subsequently confirmed as *S. parasuis*.

Regarding epidemiological exposure, the patient reported no occupational contact with live animals and no household livestock. However, she reported direct handling of raw pork products purchased from a local slaughter market shortly before symptom onset. No similar symptoms were reported among family members.

Following microbiological confirmation, public health authorities were notified, and appropriate epidemiological investigation and preventive measures were initiated. The bacterial isolate was submitted for further confirmation and genomic analysis.

## 3 Materials and methods

### 3.1 Sample collection and bacterial isolation

A clinical isolate of *S. parasuis* was obtained from the blood culture of a confirmed human case at Beijing Chaoyang Hospital, Capital Medical University, in July 2024. The isolate was collected as part of routine clinical diagnostics and used for downstream microbiological and genomic analyses. Blood culture sample were streaked onto Columbia blood agar plates (Oxoid, UK) and incubated at 37 °C under 5% CO_2_ for 24 h. After incubation, small colonies (0.5-1.0 mm in diameter) with α-hemolysis were observed. Colonies were circular, smooth, moist, and milky white in appearance. Gram staining revealed Gram-positive cocci.

The isolate was identified as *S. parasuis* using standard microbiological methods and was preserved in 25% (v/v) glycerol at −80 °C for subsequent whole-genome sequencing, comparative genomics, and molecular analyses.

### 3.2 DNA extraction and whole-genome sequencing

Genomic DNA was extracted from bacterial cultures using the TIANamp Bacterial Genomic DNA Kit (DP302, TIANGEN, Beijing, China) and further purified with the Agencourt AMPure XP Kit (Beckman Coulter, USA) following the manufacturers’ instructions. For Illumina sequencing, DNA libraries were prepared using the xGen DNA Library Preparation Kit (IDT, USA), generating 150 bp paired-end reads with a total of 757,593,900 bases and an average genome coverage of approximately 379×.

For Oxford Nanopore sequencing, 1,000 ng of high-quality genomic DNA was used for library construction following the Native Barcoding Genomic DNA protocol (Oxford Nanopore Technologies, ONT). Sequencing on a MinION flow cell generated 5,438,664 reads totaling 3,568,976,045 bases, with an average read length of 656 bp, N50 of 770 bp, and a maximum read length of 73,002 bp, providing an approximate genome coverage of 1,785×.

### 3.3 Genome assembly and annotation

Open reading frames (ORFs) and protein sequences were predicted using Prokka (version 1.14.6) (Seemann, 2014). Proteins were functionally annotated using eggNOG-mapper (version 2.1.12) (Cantalapiedra et al., 2021), based on the eggNOG 5.0 database (Huerta-Cepas et al., 2019), which integrates COG (Clusters of Orthologous Groups), GO (Gene Ontology), and KEGG (Kyoto Encyclopedia of Genes and Genomes) annotations. Annotated genome files in GenBank format were generated for downstream analyses.

Virulence factors were identified by BLASTp (version 2.12.0+) (Altschul et al., 1990) by comparison predicted proteins against the VFDB setB_pro database (downloaded on 2022-07-09) (Chen et al., 2005), using an E-value threshold of 1e-8. Antibiotic resistance genes were predicted using Resistance Gene Identifier (RGI, version 4.0.1) with the CARD database (version 4.0.1) (Alcock et al., 2023), integrating BLASTp homology search and protein domain model alignment via (DIAMOND, version 2.0.11) (Brenner, 1990) to detect potential resistance mechanisms.

### 3.4 Comparative genomics and phylogenetic analysis

Average Nucleotide Identity (ANI) analysis was performed using FastANI (version 1.33) (Jain et al., 2018) to quantify genetic relatedness between genomes. The analysis included the newly sequenced *S. parasuis* strain S62, 13 previously reported *S. parasuis* strains (1628469, SUT-28, NX1, BS26, NN1, 221006, 7500, SS17, SS20, H35, FZ2, FZ1, SFJ45), and two *S. suis* reference strains (BM4071 and NCTC10234). ANI values, calculated based on the average nucleotide similarity of shared orthologous genes or genomic fragments, provide a robust metric for microbial species delineation.

Phylogenetic relationships among strains were inferred using both the core genome and selected housekeeping genes. Homologous gene clustering was performed using Roary (version 3.12.0) (Page et al., 2015) to identify single-copy core genes shared among all strains. For the core genes as well as the 16S rRNA, *groEL*, *gyrB*, *sodA*, and *recN* genes extracted from annotated genome assemblies, were aligned using MUSCLE (version 3.8.31) (Edgar, 2004). Alignments were converted into PHYLIP format and used to calculate pairwise genetic distances with the dnadist program in PHYLIP (version 3.697) (Retief, 2000), applying the Kimura 2-parameter model (transition/transversion ratio set to 2.0) with interleaved input enabled.

Phylogenetic trees were reconstructed using the Neighbor-Joining (NJ) method. For 16S rRNA trees, *Enterococcus faecalis* JCM 5803 was designated as the outgroup; for core genome and other housekeeping gene trees, *S. suis* NCTC10234 served as the outgroup. Node reliability was assessed with 1,000 bootstrap replicates and summarized using the Majority-rule extended (MRE) consensus method. Bootstrap values ≥90%, 70–89%, and <70% were considered strong, moderate, and weak, respectively. The resulting trees were visualized and annotated using the Interactive Tree Of Life (iTOL) tool (Letunic and Bork, 2024).

### 3.5 Antimicrobial susceptibility testing

Antimicrobial susceptibility of *S. parasuis* strain S62 was determined using the broth microdilution method (Fosun Diagnostics, Changsha, China), following CLSI M100 34th Edition guidelines. Tested antibiotics included penicillin, amoxicillin, meropenem, levofloxacin, tetracycline, clindamycin, chloramphenicol, linezolid, vancomycin, teicoplanin, and cefepime. Inocula were prepared from overnight cultures, adjusted to 0.5 McFarland standard, and incubated at 37 °C for 24 h in 5% CO_2_. Quality control was performed using *Streptococcus pneumoniae* ATCC 49619, and MIC values were interpreted according to CLSI species-specific breakpoints.

## 4 Results

### 4.1 Genome assembly

The genome of the *S. parasuis* isolate was assembled into a total length of 1,933,868 bp with a G+C content of 39.50%. Assembly statistics indicated that the largest contig reached 342,011 bp, while the N50 value was 123,522 bp (L50 = 4). The average contig length of N90 was 32,777.42 bp, with the N90 contig length being 30,184 bp (L90 = 14). The minimum contig length was 222 bp. Contig counts for sequences longer than 500 bp and 10 kb were 38 and 21, respectively, indicating a high continuity of the assembled genome.

### 4.2 Average nucleotide identity analysis

**Figure 1:**
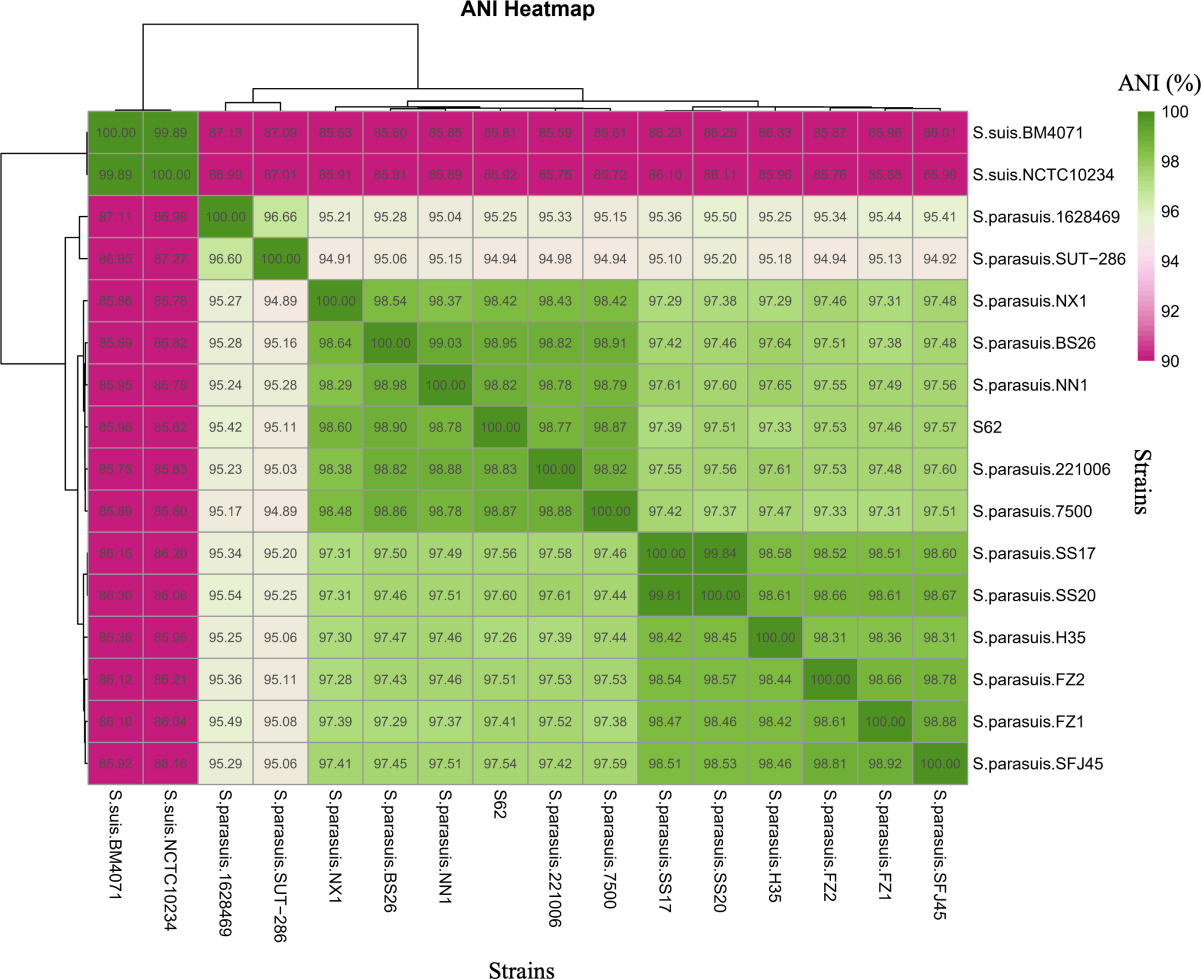
Heatmap of Average Nucleotide Identity (ANI) values among *S. parasuis* S62, other *S. parasuis* strains, and *S. suis* reference strains.

ANI analysis was performed to clarify the taxonomic status of the isolate S62. The ANI values between S62 and other *S. parasuis* strains ranged from 97.0% to 98.9%, with the highest similarity observed with *S. parasuis* BS26 (98.9%). By contrast, ANI values between S62 and *S. suis* reference strains (BM4071 and NCTC10234) were markedly lower, ranging from 85.8% to 85.9%. The observed ANI values place S62 well above the commonly accepted species-level threshold for S. parasuis and clearly below that for S. suis. The ANI heatmap further illustrates high intra-species similarity among *S. parasuis* isolates and a clear separation from *S. suis* genomes (**Figure 1**).

### 4.3 Identification and sequence analysis of virulence genes

Virulence-associated genes in strain S62 were screened using the Virulence Factor Database (VFDB) combined with comparative genomic analyses. Among the established virulence determinants of *S.* spp., the *hasC* gene was identified in the genome of strain S62. This gene was located between positions 1,054,330 and 1,055,205 bp, with an alignment coverage of 95.6% and sequence identity of 75.9% compared to the reference sequence (accession number NP_270109). The *hasC* gene encodes UTP--glucose-1-phosphate uridylyl transferase HasC, which participates in capsular polysaccharide biosynthesis and has been implicated in bacterial pathogenicity (Wessels, 2019).

In contrast, several classical *S. suis* virulence factors, including *mrp*, *epf*, *sly*, and the capsule synthesis gene cluster (cps), were not detected in strain S62. In particular, the commonly reported adhesion-associated gene *srtA* was absent. Although a weak match to *S. sanguinis srtA* was observed (57% coverage and 78% sequence identity), this partial similarity does not represent a functional homolog in *S. parasuis*. Overall, the virulence gene profile of strain S62 differs from that of typical *S. suis* reference strains.

Given that approximately 20.5% of the predicted protein-coding genes in strain S62 were annotated as hypothetical proteins, further homology-based analyses were conducted to assess their potential biological relevance. Among the 700 hypothetical proteins, 663 showed significant homology to sequences in the nr database, indicating that most of these proteins are not unique to strain S62. Taxonomic analysis of homologous hits revealed that 380 hypothetical proteins were most closely related to members of the genus *Streptococcus*, including *S. suis* (150), *S. parasuis* (139), and other related species such as *S. equinus*, *S. ruminantium*, and *S. thermophilus*. An additional 126 homologs were annotated as originating from *Bacilli bacterium*, reflecting conserved proteins shared among closely related taxa within the class *Bacilli*.

Functional annotation of the homologous proteins indicated that most were associated with core cellular processes, including metabolism, transport, and genetic information processing. No clear enrichment of known virulence-associated domains or host-interaction motifs was identified among the hypothetical protein set.

### 4.4 Antibiotic resistance genes detection and potential resistance analysis

Antibiotic resistance genes in strain S62 were screened using the Comprehensive Antibiotic Resistance Database (CARD). Three resistance-associated genes were identified. A putative homolog of *vanY* was detected and annotated as *vanY_in_vanB_cl* (ARO:3002956), a variant of *vanY* typically found within the *vanB* glycopeptide resistance cluster (Courvalin, 2006). The best hit shared ~42.9% amino acid identity with the reference sequence (bitscore ≈ 92.0). According to CARD, *vanY_in_vanB_cl* belongs to the glycopeptide resistance gene family and mediates resistance through antibiotic target alteration, with perfect matches reported in *Enterococcus* spp (Evers and Courvalin, 1996). However, the *vanY*-like sequence detected in strain S62 showed relatively low similarity to the reference.

Phenotypic antimicrobial susceptibility testing indicated that strain S62 was susceptible to glycopeptides, with MIC values of 8 µg/mL for vancomycin and ≤0.5 µg/mL for teicoplanin. These results suggest that the *vanY*-like homolog identified in S62 is not associated with detectable glycopeptide resistance under the tested conditions.

In addition, two ABC transporter genes, *patA* (ARO:3000024) and *patB* (ARO:3000025), were identified. These genes are known to encode a paired efflux pump system in *Streptococcus* species and have been implicated in reduced susceptibility to fluoroquinolones (El Garch et al., 2010). Both genes in S62 showed high similarity to their respective CARD references and were annotated as components of the ATP-binding cassette (ABC) antibiotic efflux pump family. The *patA–patB* system has been reported with sequence variants across multiple *Streptococcus* species, including *S. suis* (Garvey et al., 2011).

Antimicrobial susceptibility testing showed that strain S62 was susceptible to levofloxacin (MIC = 7 µg/mL). Intermediate susceptibility was observed for tetracycline (MIC = 5 µg/mL) and clindamycin (MIC = 0.5 µg/mL). The isolate remained susceptible to all other tested antibiotics, including penicillin, amoxicillin, meropenem, chloramphenicol, linezolid, and cefepime.

### 4.5 Functional annotation

**Figure 2:**
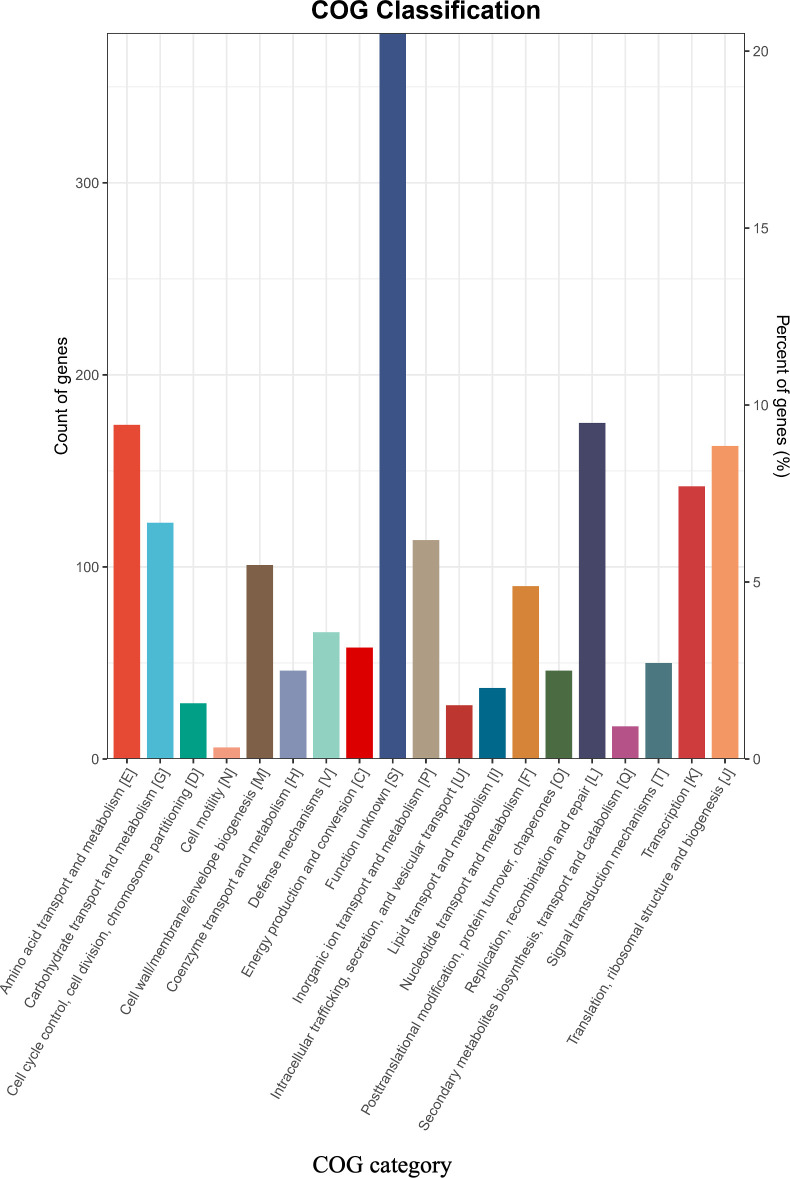
Cluster of orthologous groups (COG) functional classification of *S. parasuis* S62 genome.

Functional annotation of the *S. parasuis* S62 genome was performed using COG, GO and KEGG databases to investigate its functional potential. Based on COG classification, 1,843 genes were assigned to 19 functional categories. The largest proportion was “function unknown” (20.5%), followed by replication, recombination and repair (9.5%), amino acid transport and metabolism (9.4%), translation, ribosomal structure and biogenesis (8.8%), and transcription (7.7%). Other notable categories included carbohydrate transport and metabolism (6.7%), inorganic ion transport and metabolism (6.2%), and cell wall/membrane/envelope biogenesis (5.5%) (**Figure 2**).

**Figure 3:**
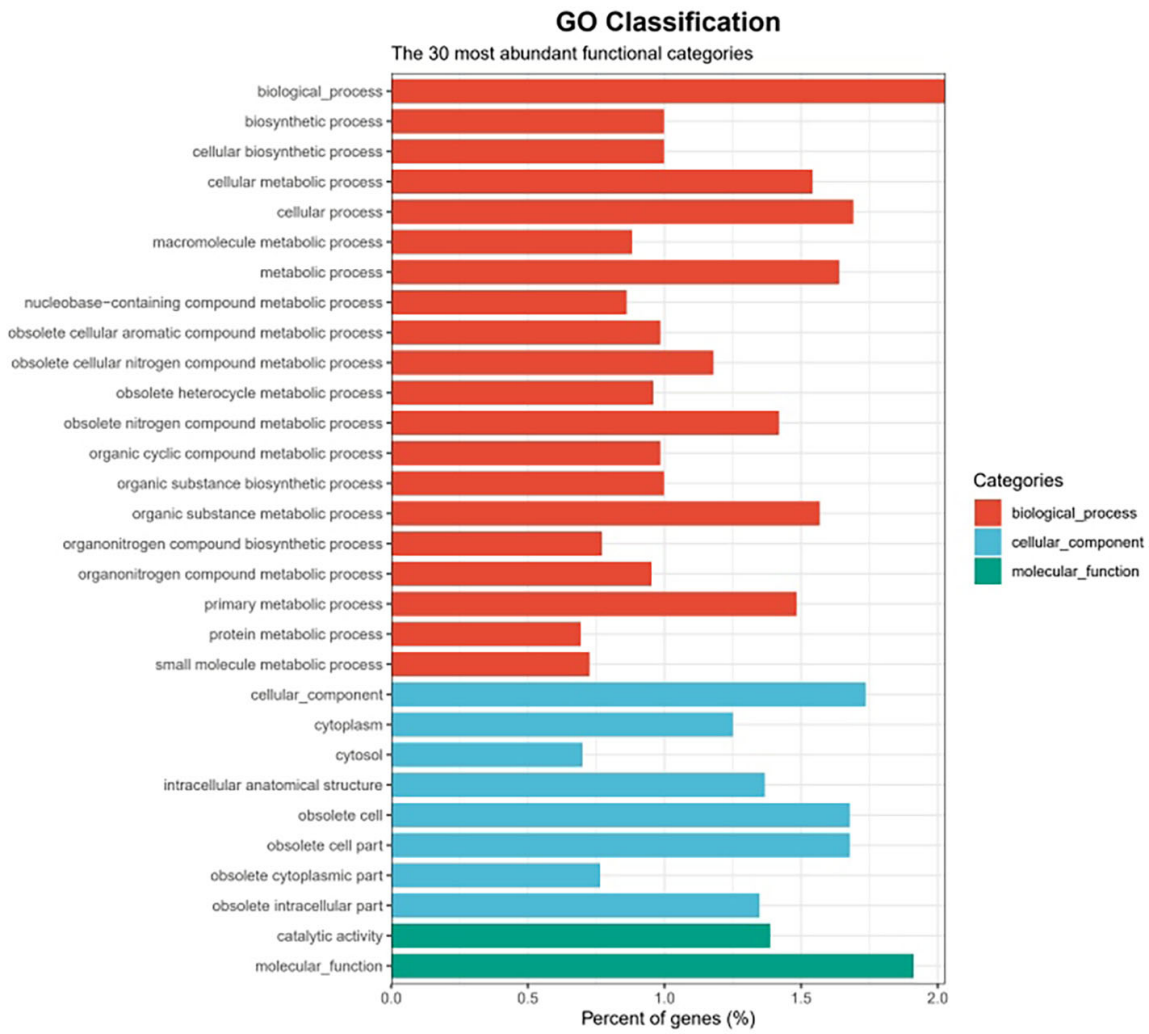
Gene ontology (GO) functional classification of *S. parasuis* S62 genome. Genes were annotated into three major categories (biological process, cellular component, and molecular function), and the top 30 subcategories are shown.

GO annotation assigned 15,432 GO entries and grouped them into three primary categories: biological process (64.6%), cellular component (16.8%) and molecular function (18.6%). At the secondary (GO level-2) level, there were 1,686 distinct terms; the top 30 level-2 categories are shown in **Figure 3**. Among these, the most abundant secondary terms were: the leading biological process term (313 entries, 2.02% of all GO annotations), the leading molecular function term (295 entries, 1.91%), and the leading cellular component term (268 entries, 1.73%).

**Figure 4:**
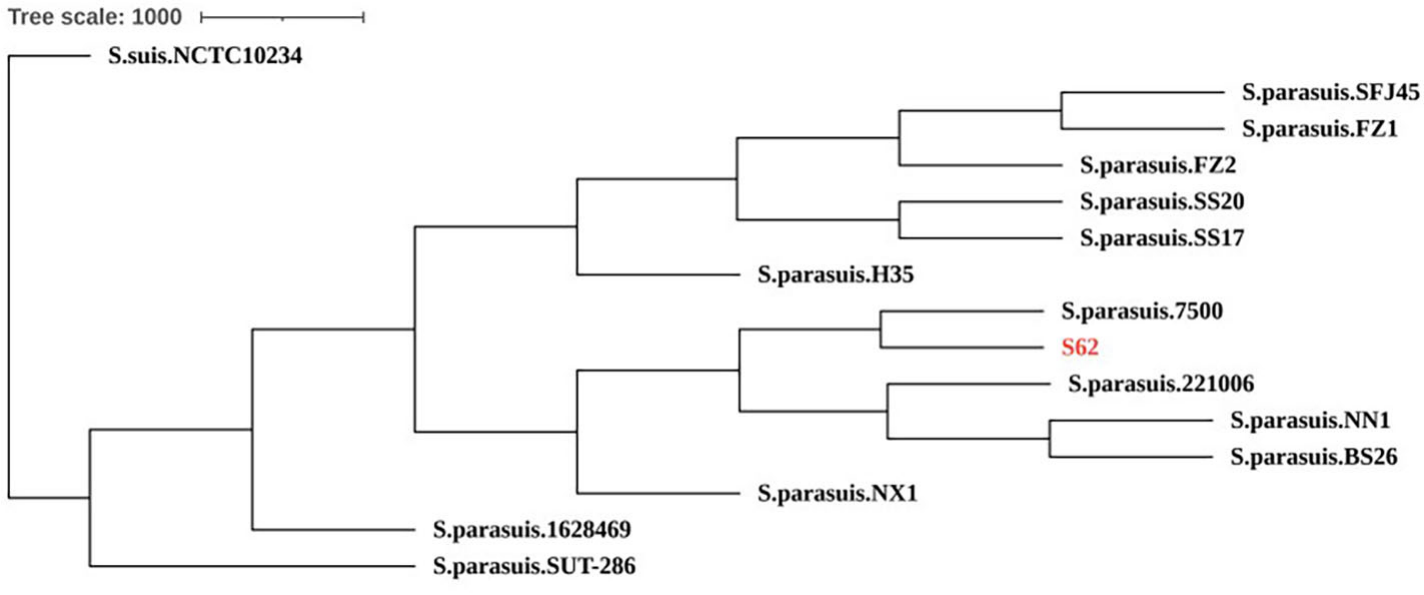
Kyoto Encyclopedia of Genes and Genomes (KEGG) pathway annotation of *S. parasuis* S62 genome. Genes were assigned to six major pathway categories, and the top 30 subcategories are displayed.

KEGG pathway annotation mapped 1,380 genes to pathways grouped into six primary categories: Cellular Processes (6.38%), Environmental Information Processing (13.33%), Genetic Information Processing (13.48%), Human Diseases (5.29%), Metabolism (59.06%), and Organismal Systems (2.46%). At KEGG subcategories, there are 36 pathway classes, and the top 30 by gene count are displayed in **Figure 4**. The most represented subcategories pathways were carbohydrate metabolism (16.38%), amino acid metabolism (12.68%), membrane transport (9.42%), and nucleotide metabolism (7.75%).

### 4.6 Phylogenetic analysis based on single-gene and whole-genome sequences

**Figure 5:**
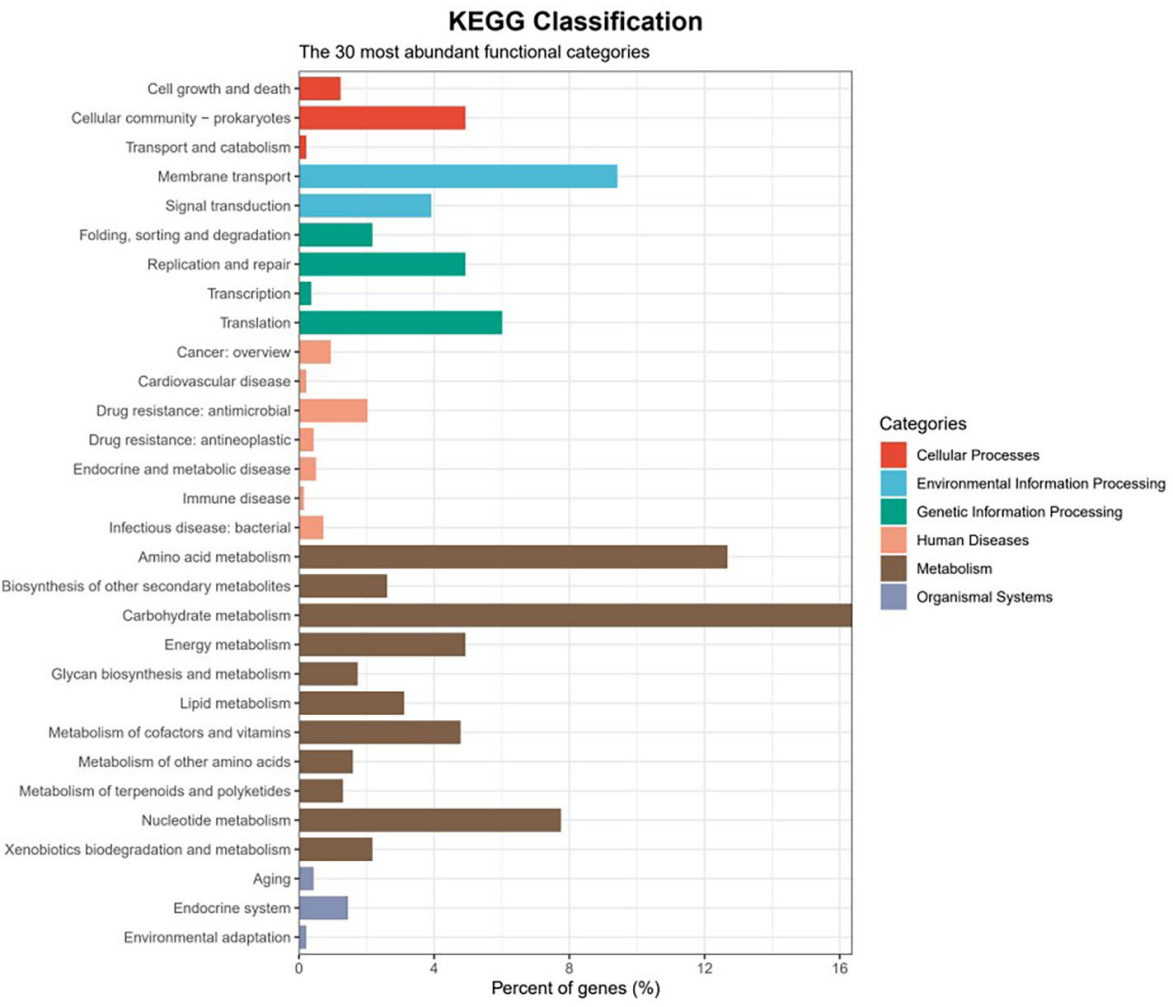
Phylogenetic trees of *S. parasuis* S62 based on individual housekeeping genes: **(A)** 16S rRNA, **(B)** *groEL*, **(C)** *gyrB*, **(D)** *sodA*, and **(E)** *recN*. *E. faecalis* JCM 5803 was used as the outgroup for 16S rRNA, and *S. suis* NCTC10234 for the other genes.

Phylogenetic analyses were performed using both five housekeeping genes (16S rRNA, *groEL*, *gyrB*, *recN*, and *sodA*) and the concatenated single-copy core genes to determine the evolutionary position of strain S62 among *S. parasuis* and *S. suis* strains. Analyses based on individual housekeeping genes revealed generally similar phylogenetic patterns, although minor topological differences were observed among loci (**Figures 5A–E**).

In the 16S rRNA tree (**Figure 5A**), S62 clustered with NX1 (bootstrap = 1000), whereas *groEL* (**Figure 5B**) grouped S62 together with NN1, BS26, and NX1 (bootstrap = 1000). The *gyrB* phylogeny (**Figure 5C**) placed S62 closest to 7500 and NN1 (bootstrap = 1000), the *recN* tree (**Figure 5D**) positioned it adjacent to the clade containing 221006, 7500, NN1, NX1, and BS26 (bootstrap = 1000), and in the *sodA* tree (**Figure 5E**), S62 was most closely related to NX1 and BS26 (bootstrap = 1000). For the 16S rRNA tree, *E. faecalis* JCM 5803 was used as the outgroup, whereas for *S. suis* strain NCTC10234 was used as the outgroup for the remaining housekeeping gene trees.

**Figure 6:**
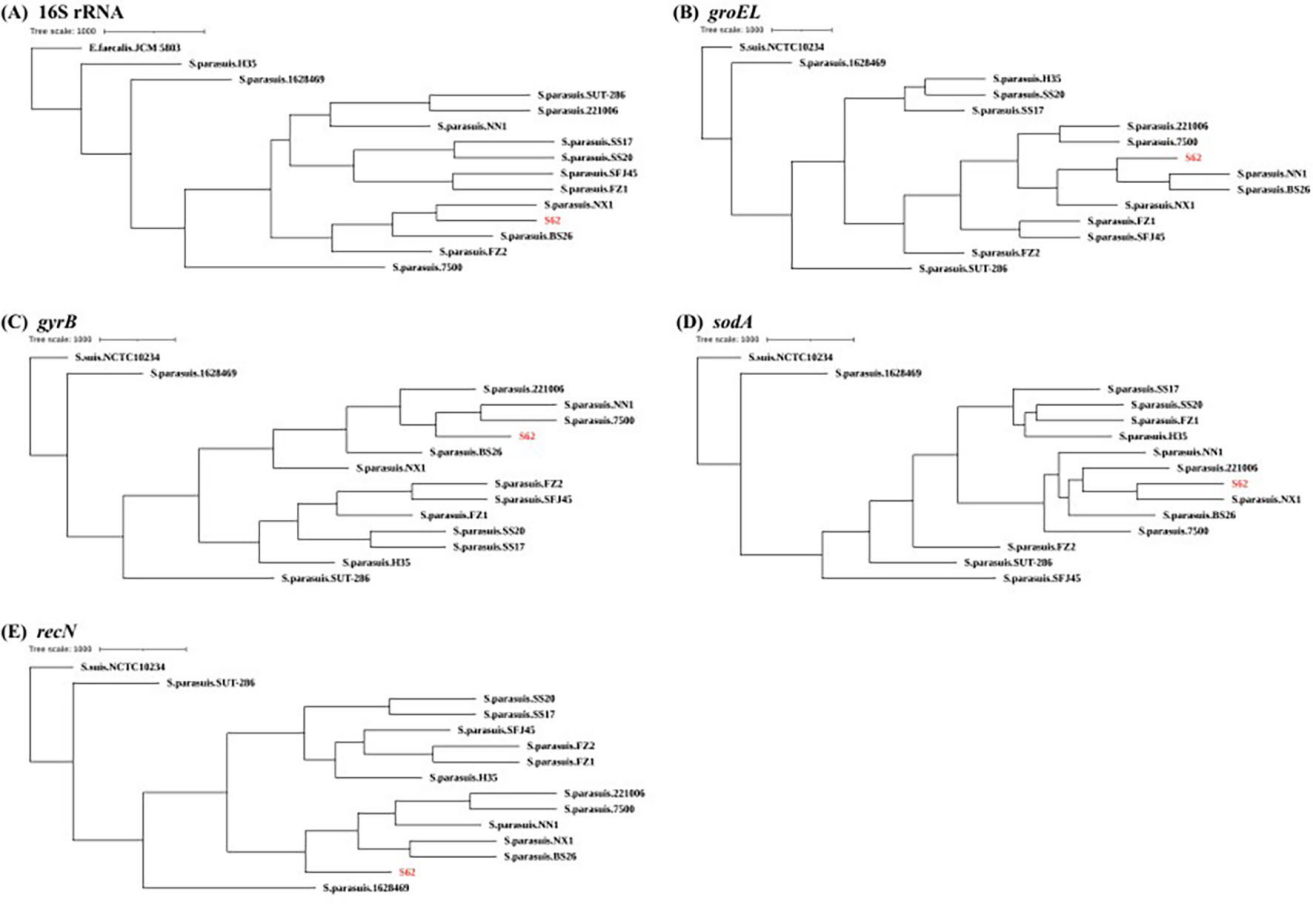
Whole-genome phylogenetic tree of *S. parasuis* S62 and related strains based on 454 single-copy core genes. *S. suis* NCTC10234 was used as the outgroup. Panels correspond to individual gene phylogenies: **(A)** 16S rRNA, **(B)** groEL, **(C)** gyrB, **(D)** sodA, and **(E)** recN. Isolate S62 is highlighted in red text in all trees.

The core genome phylogeny, inferred from 454 single-copy core genes shared among all strains (**Figure 6**), placed S62 firmly within the *S. parasuis* clade, distinct from the outgroup. Strain S62 clustered most closely with strain 7500, forming a strongly supported subclade (bootstrap = 1000), and was also closely related to strains 221006, NN1, BS26, and NX1. Notably, strain 7500 was originally isolated from diseased pigs in China (GenBank NZ_CP128410).

Together, phylogenetic analyses based on both individual housekeeping genes and concatenated single-copy core genes consistently placed strain S62 within the *S. parasuis* clade, clearly separating it from *S. suis* reference strains (BM4071 and NCTC10234), while resolving intra-species relationships among *S. parasuis* isolates.

## 5 Discussion

This study reports a rare human infection caused by *S. parasuis* and provides a comprehensive genomic characterization of the clinical isolate S62. To date, human cases of *S. parasuis* infection remain uncommon, and most existing knowledge regarding this species is derived from animal isolates, particularly from pigs. The present case therefore expands the clinical spectrum of *S. parasuis* infection in humans and underscores its potential relevance as an emerging zoonotic pathogen. The patient presented with recurrent febrile illness and underlying metabolic comorbidity, which may have increased susceptibility to infection, consistent with previous observations that opportunistic infections by animal-associated streptococci often occur in immunocompromised or metabolically compromised hosts.

Genomic analysis revealed that strain S62 harbors a limited but conserved repertoire of virulence-associated genes. Among established streptococcal virulence determinants, *hasC*, which is involved in capsular polysaccharide biosynthesis, was identified and may contribute to immune evasion and persistence within the host. In contrast, several classical *S. suis* virulence factors, including *mrp*, *epf*, *sly*, and *srtA*, were absent. Notably, the absence of *srtA*, which mediates the surface anchoring of multiple virulence-associated proteins in streptococci, has also been reported in several animal-derived *S. parasuis* isolates. This suggests that *S. parasuis* may employ virulence strategies distinct from those of highly pathogenic *S. suis* strains, potentially relying more on capsule-associated mechanisms rather than surface-anchored adhesins or cytotoxins. Furthermore, although a substantial proportion of the genome was initially annotated as hypothetical proteins, homology-based analyses indicated that most of these genes are conserved among *Streptococcus* species and are primarily associated with core cellular functions rather than novel virulence traits. Together, these features are consistent with a moderate pathogenic potential, which may partly explain the rarity of severe or invasive human infections caused by *S. parasuis*.

Antimicrobial resistance profiling demonstrated a general concordance between genomic predictions and phenotypic susceptibility testing. Although a *vanY*-like homolog and the *patA–patB* efflux system were detected in the genome, strain S62 remained susceptible to glycopeptides and fluoroquinolones under the tested conditions. These observations highlight the importance of cautious interpretation of resistance gene annotations, as the presence of resistance-associated genes does not necessarily translate into clinically relevant resistance. The largely susceptible phenotype observed in this isolate suggests that current first-line antimicrobial agents remain effective for treating human *S. parasuis* infections, while also emphasizing the value of integrating genomic data with phenotypic validation.

Phylogenetic analyses based on both single housekeeping genes and core genome sequences consistently placed strain S62 within the *S. parasuis* clade and revealed a close genetic relationship with several animal-derived isolates, including strain 7500 isolated from diseased pigs in China. While this phylogenetic proximity raises the possibility of an animal origin, genetic relatedness alone is insufficient to infer transmission events. In the present study, no epidemiological link to live pigs was identified, exposure was limited to retail pork products, and no sampling or sequencing of the suspected food source or its supply chain was performed. Moreover, no geographic or temporal overlap between strain S62 and closely related animal isolates could be demonstrated. These limitations preclude definitive conclusions regarding cross-species transmission. Future studies incorporating systematic surveillance, source tracing, and comparative genomics across human, animal, and food-associated isolates will be essential to clarify the zoonotic potential and transmission dynamics of *S. parasuis*.

From a clinical and diagnostic perspective, *S. parasuis* may be easily misidentified as *S. suis* in routine diagnostic laboratories due to their close phylogenetic relationship and overlapping phenotypic characteristics. This underscores the importance of accurate species identification using genome-based approaches, particularly as human infections caused by non-*S. suis* streptococci may be underrecognized. Improved genomic surveillance and diagnostic resolution will be critical for understanding the true clinical burden and epidemiology of *S. parasuis* infections.

## 6 Conclusions

We report the isolation and genomic characterization of a clinical *S. parasuis* strain S62 from a febrile patient. Genome sequencing and comparative analyses confirmed that S62 belongs to *S. parasuis*, clearly distinct from *S. suis*, and revealed conserved virulence-associated features, including the capsular polysaccharide biosynthesis gene *hasC*, as well as resistance-related elements such as a vanY-like gene and the *patA–patB* efflux system.

Genome sequencing and comparative analyses confirmed that S62 belongs to *S. parasuis*, clearly distinct from *S. suis*, and revealed conserved virulence-associated features, including the capsular polysaccharide biosynthesis gene *hasC*, as well as resistance-related elements such as a vanY-like gene and the *patA–patB* efflux system. Together, these findings provide the first comprehensive genomic and phenotypic characterization of a human-derived *S. parasuis* isolate in Beijing. This study establishes baseline data for surveillance of emerging *S. parasuis* infections and supports further investigations into its cross-species transmission potential, pathogenic mechanisms, and clinical significance.

## Data Availability

The sequencing data generated in this study have been deposited in the Genome Sequence Archive (GSA) at the China National Center for Bioinformation (CNCB, https://ngdc.cncb.ac.cn/gsa) under the accession ID CRA031336.
